# Associations between adverse childhood experiences and early adolescent problematic screen use in the United States

**DOI:** 10.1186/s12889-023-16111-x

**Published:** 2023-06-22

**Authors:** Julia H. Raney, Abubakr. A Al-shoaibi, Kyle T. Ganson, Alexander Testa, Dylan B. Jackson, Gurbinder Singh, Omar M. Sajjad, Jason M. Nagata

**Affiliations:** 1 Department of Pediatrics, Division of Adolescent and Young Adult Medicine, University of California, San Francisco, CA USA; 2 Factor-Inwentash Faculty of Social Work, University of Toronto, Toronto, ON Canada; 3 Department of Management, Policy and Community Health, University of Texas Health Science Center at Houston, Houston, TX USA; 4 Department of Population, Family and Reproductive Health, Johns Hopkins Bloomberg School of Public Health, Baltimore, MD USA; 5 Geisel School of Medicine, Dartmouth College, Hanover, NH USA

**Keywords:** Social media, Video game, Adolescents, Adolescent brain cognitive development study

## Abstract

**Background:**

Problematic screen use, defined as an inability to control use despite private, social, and professional life consequences, is increasingly common among adolescents and can have significant mental and physical health consequences. Adverse Childhood Experiences (ACEs) are important risk factors in the development of addictive behaviors and may play an important role in the development of problematic screen use.

**Methods:**

Prospective data from the Adolescent Brain Cognitive Development Study (Baseline and Year 2; 2018–2020; N = 9,673, participants who did not use screens were excluded) were analyzed in 2023. Generalized logistic mixed effects models were used to determine associations with ACEs and the presence of problematic use among adolescents who used screens based on cutoff scores. Secondary analyses used generalized linear mixed effects models to determine associations between ACEs and adolescent-reported problematic use scores of video games (Video Game Addiction Questionnaire), social media (Social Media Addiction Questionnaire), and mobile phones (Mobile Phone Involvement Questionnaire). Analyses were adjusted for potential confounders including age, sex, race/ethnicity, highest parent education, household income, adolescent anxiety, depression, and attention-deficit symptoms, study site, and participants who were twins.

**Results:**

The 9,673 screen-using adolescents ages 11–12 years old (mean age 12.0) were racially and ethnically diverse (52.9% White, 17.4% Latino/Hispanic, 19.4% Black, 5.8% Asian, 3.7% Native American, 0.9% Other). Problematic screen use rates among adolescents were identified to be 7.0% (video game), 3.5% (social media), and 21.8% (mobile phone). ACEs were associated with higher problematic video game and mobile phone use in both unadjusted and adjusted models, though problematic social media use was associated with mobile screen use in the unadjusted model only. Adolescents exposed to 4 or more ACEs experienced 3.1 times higher odds of reported problematic video game use and 1.6 times higher odds of problematic mobile phone use compared to peers with no ACEs.

**Conclusions:**

Given the significant associations between adolescent ACE exposure and rates of problematic video and mobile phone screen use among adolescents who use screens, public health programming for trauma-exposed youth should explore video game, social media, and mobile phone use among this population and implement interventions focused on supporting healthy digital habits.

**Supplementary Information:**

The online version contains supplementary material available at 10.1186/s12889-023-16111-x.

## Introduction

Problematic screen use has risen dramatically among adolescents; 45% of adolescents report being online “almost constantly.” [1] Screen use becomes problematic when the user experiences a loss of control over usage and impairments in personal, social, and occupational functioning [2]. More specifically, we define problematic screen use as demonstrating key elements of the six core components of behavioral addiction: salience (the activity dominates thinking), mood modification (the activity impacts mood), tolerance (increasing time spent on the activity is needed to achieve previous effects), withdrawal (mood worsens when the activity is discontinued or reduced), conflict (the activity negatively impacts relationships), and relapse (a pattern of returning to use following a period of abstinence or improved control) [3]. These problematic use patterns can span a variety of modalities, including video games, social media, and phones [2, 4–6]. Problematic video game use is characterized by a sense of euphoria while playing, inability to stop, craving more time, low mood when not playing, and consequences in private, social and professional life [2]. Problematic social media use is characterized by an internalized need to be constantly connected via technology [5, 7]. Problematic mobile phone use includes a broader range of applications (i.e. texting, apps, video chat) but shares the same behavioral characteristics as those described above [6]. Given that excessive screentime and screen addictions are associated with reductions in physical activity [8], increased risk of obesity [5, 9], and psychological consequences including obsessive-compulsive disorder (OCD), anxiety, and depression [2, 5–7, 10], identifying risk factors to inform prevention and intervention efforts is critical.

Recent research has highlighted that adverse childhood experiences (ACEs), defined as potentially traumatic events that occur before the age of 18, are an important risk factor in developing addictive behaviors [11, 12]. Several studies have shown that adolescents who have experienced childhood trauma have a higher risk of developing problematic video game [13, 14], internet [15–17], and mobile phone use [18]. However, few studies have explored this relationship in a large, nationally representative U.S. sample in the setting of recent screen use increases. Further, few studies have used youth self-report screen-time data or focused on early adolescents, an age range when a spike in computer use, gaming, and social media use often occurs [19]. Moreover, the relationship between ACEs and problematic social media use has yet to be explored. This study aims to fill these gaps by examining the associations between ACEs and problematic video game, social media, and mobile phone use among a large, nationally representative sample of U.S. early adolescents. It is hypothesized that higher adolescent ACE scores will be associated with higher rates of problematic video game, social media, and phone use among youth who report screen use.

## Methods

This study used a prospective design to determine the association between ACE score and problematic screen use among U.S. early adolescents. This study used survey data from the Adolescent Brain Cognitive Development (ABCD) study, a large, diverse, prospective cohort study of brain development and health among adolescents from 21 recruitment sites across the U.S [20]. To maximize retention, research staff connects with families at least every six months by telephone and every year in person. To prevent higher attrition rates from lower-income families, the study provides a free nutrition and exercise program, a meal, homework assistance, childcare for other family members who accompanied the participant to the visits, and transportation vouchers during research visits. Youth who did not participate in any type of screen use (video game, social media, or mobile phone, were excluded (n = 2,112) The final sample consisted of 9,763 adolescents ages 11–12 years old during the two-year follow-up (4.0 release). Centralized institutional review board (IRB) approval was received from the University of California, San Diego (UCSD). Written informed consent and assent were obtained from caregivers and the child, respectively.

### Measures

#### Exposure variables

ACE score was calculated through adolescent and parent responses from the baseline (2016–2018) survey. The ABCD study assesses nine of ten ACEs reflecting the items in the original CDC-Kaiser ACE study across different surveys as a validated ACEs screener was not administered. This generated scale has been used in prior ABCD literature and is based Hoffman et al.’ recommendations [21–24]. Supplemental Table 1 highlights how these questions map onto validated ACEs questions. Emotional abuse was not assessed in the ABCD study and therefore not included. ACE score was then categorized as 0 through ≥ 4, as a cumulative ACE score of ≥ 4 has documented greater risk concentration at this threshold [25, 26].

#### Outcome variables

Adolescents completed the following questionnaires at the two-year follow-up (4.0 release), the first time these surveys were administered.

The **Video Game Addiction Questionnaire (VGAQ)** is a six-question instrument used to assess problematic video game use. The questions were adapted from the Bergen Facebook Addiction Scale [27]. The Bergen Facebook Addiction Scale consists of a single factor structure questionnaire assessing the six core components of behavioral addiction (salience, mood modification, tolerance, withdrawal, conflict, relapse) and has been validated in numerous different clinical and cultural contexts [28–30], but authors have previously extrapolated its application to broader video game addiction among early adolescents and college students [4, 31]. Questions assessing components of addiction include “I spend a lot of time thinking about playing video games” and “I’ve become stressed or upset if I am not allowed to play video games.” Likert-type scale responses ranged from 1 (never) to 6 (very often). Participants who reported any video game use on weekdays or weekends were asked these items. Responses were averaged from 0 to 6. If a response was missing, the average was calculated based on available responses. Participants who reported no use were excluded. Of note, 99.5% of participants completed the entire subscale once started. In this study, the Cronbach’s alpha was 0.85, mean 2.1, SD 1.1, and range 1–6. A cutoff score of 4 or greater was characterized as problematic video game use based on conservative suggestions from the Bergen Facebook Addition Scale literature [27, 32].

The **Social Media Addiction Questionnaire (SMAQ)** is a six-question survey also adapted from the Bergen Facebook Addiction Scale that assesses the six aforementioned components of behavioral addiction [33]. The SMAQ has been extrapolated to problematic social media use among early adolescent, high school, and college students [4, 31, 34, 35]. Example questions include “I feel the need to use social media apps more and more” and “I use social media apps so much that it has had a bad effect on my schoolwork or job.” Likert-type scale responses ranged from 1 (never) to 6 (very often). Participants who reported having at least one social media account were asked these items. Participants who reported no use were excluded. In this study, the Cronbach’s alpha was 0.82, mean 1.8, SD 0.9, and range 1–6. As above, a cutoff score of 4 or greater was classified as problematic social media use [32, 33].

The eight question **Mobile Phone Involvement Questionnaire (MPIQ)** is an 8-item survey developed to assess problematic phone use in adolescents and also measures the core components of behavioral addiction including salience, euphoria, withdrawal, and tolerance [36]. This instrument has been previously used in a study of U.S. high school students examining smartphone dependence [37]. Examples include “I lose track of how much I am using my phone” and “Arguments have arisen because of my phone use.” Likert-type scale responses ranged from 1 (strongly disagree) to 7 (strongly agree). Participants who reported having mobile phones were asked these items. Responses were averaged from 0 to 7. Participants who reported no use were excluded. In this study, the Cronbach’s alpha was 0.79, mean 3.1 SD 1.1, and range 1–7. Based on prior literature, scores of four or greater were considered problematic mobile phone use [38].

### Covariates

Parents reported participants’ age, sex (male or female) and race/ethnicity (White, Black, Native American, Latino/Hispanic, Asian, or Other) at baseline. Parents also reported highest parent education (high school or lower versus college or higher) and household income (less than $25,000, $25,000 - $50,000, $50,000 - $75,000, $75,000 - $100,000, $100,000 - $200,000, and greater than $200,000) at baseline Depressive, anxious, and attention-deficit symptoms at baseline were generated from parent/caregiver responses to the Child Behavior Checklist (CBCL), a screening tool that asks a parent/caretaker about multiple psychiatric symptoms and behavior problems in children ages 4–18 [20, 39]. We included t scores of depressive, anxious, and attention-deficit scales from the CBCL. CBCL raw scores for each scale were converted to norm-referenced *t*-scores (mean = 50, standard deviation 10). Separate norms were provided for gender across age groups [40]. These psychiatric symptoms were included because extensive literature establishes the relationship between ACEs and adolescent depression, anxiety, and ADHD [41, 42], and these mental health conditions have been associated with higher levels of problematic screen use [43] and have been included in similar analyses [17, 44]. Study site and twins were noted.

### Statistical analyses

Generalized logistic mixed effects models estimated prospective associations between baseline ACE score and problematic video game, social media, and mobile phone use (binary outcomes) among youth who reported that type of screen use. In our supplemental analyses, generalized linear effects models estimated prospective associations between baseline ACE score and problematic use scores. To account for missing data in both models, we performed multiple imputation by chained equations using the R package “mice”. There were missing data including twins, 3 (0.04%); gender, 5 (0.07%); race, 33 (0.4%); depression, 1955 (25.7%); anxiety, 1955 (25.7%); and ADHD, 1955 (25.7%). Because our primary interest is the point estimates, we imputed 10 datasets and pooled the estimates from each dataset [45–47]. Supplemental Fig. 1 highlights adolescent represents included in each analysis. The supplemental analysis estimates prospective associations with continuous problematic screen use scales. Both analyses were adjusted for potential confounders listed above, accounting for study cite and twins Analyses were conducted using R version 4.2.2. Given the potential overlap between outcome variables, a correlation matrix is shown in the appendix (Supplemental Table 2).

## Results

The sample of 9,673 adolescents was racially and ethnically diverse (52.9% White, 17.4% Latino/Hispanic, 19.4% Black, 5.8% Asian, 3.7% Native American, 0.9% Other; Table 1). Participants mean age was 12.0 years old (SD 0.7, range 10.6–14). Among adolescents who used screens, 7% reported problematic video game use, 3.5% reported problematic social media use, and 21.8% reported problematic mobile phone use.

**Table 1:** Descriptive characteristics of participants in the Adolescent Brain Cognitive Development Study (ABCD) Study, 2018–2020 (N = 9,673)

	Mean (SD) or %	
**Sociodemographic characteristics**		
Age		12.0 (0.7)
Sex		
Female		46.7%
Male		53.3%
Race/ethnicity		
White		52.9%
Latino / Hispanic		17.4%
Black		19.4%
Asian		5.8%
Native American		3.7%
Other		0.9%
Highest parent education		
College education or more		86.0%
High school education or less		14.0%
Household income		
Less than $25,000		14.0%
$25,000 - $50,000		15.3%
$50,000 - $75,000		14.7%
$75,000 - $100,00		16.8%
$100,00 - $200,00		28.9%
Greater than $200,000		10.4%
Depressive problems		53.7 (5.9)
Anxiety problems		53.4 (5.0)
Attention deficit problems		53.1 (5.3)
**Self-reported adverse childhood experiences**		
No ACEs		17.6%
1 ACE		32.7%
2 ACEs		26.9%
3 ACEs		14.8%
4 or more ACEs		8:0%
**Problematic screen use measures**		
Video game use (Videogame Addiction Questionnaire) score		2.1 (1.1)a
Social media use (Social Media Addiction Questionnaire) score		1.8 (0.9)b
Mobile phone use (Mobile Phone Involvement Questionnaire) score		3.1 (1.1)c
Problematic video game use*		7.0%
Problematic social media use*		3.5%
Problematic mobile phone use*		21.8%

ACEs = Adverse Childhood Experiences

^a^Asked among a subset who reported video game use (n = 7,600)

^b^Asked among a subset who reported social media use (n = 5,656)

^c^Asked among a subset who reported mobile use (n = 7,367)

*Mean score ≥ 4

**Fig. 1:**
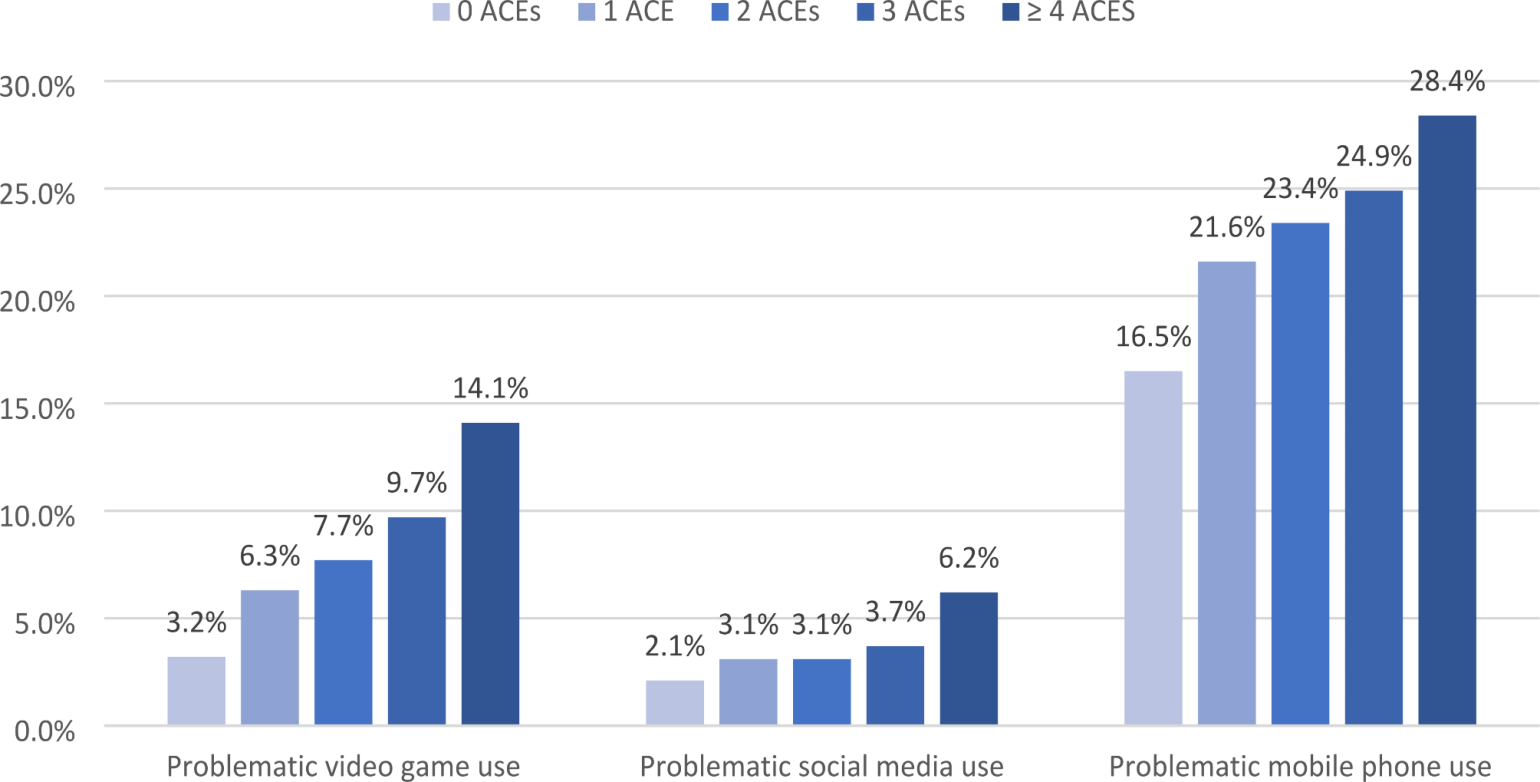
Problematic screen use (video game, social media, mobile phone)* by reported ACE score. *Problematic use characterized by binary cutoffs where mean score ≥ 4

In both the unadjusted and adjusted models, among youth who reported screen use, a higher ACE score was associated with a higher odds of problematic video game and mobile phone use. In the adjusted models, an ACE score of four or more was associated with 3.1 and 1.6 times higher odds of having problematic video game use and mobile phone use, respectively ACE score was associated with problematic video game use in a dose dependent manner. Figure 1 highlights how increasing ACE score was associated with greater rates of problematic video game and mobile phone use (Table 2). There was no statistical association between problematic social media use and ACE score. However, a higher ACE score was also associated with higher survey scores of problematic video game, media, and phone use(Supplemental Tables 3, Supplemental Fig. 2).

**Table 2:** Associations among ACE score and problematic video game use, social media use, and phone use (N = 9,673)

Panel A: Bivariate Model			
Problematic screen use measures	Problematic Video Game Use	Problematic Social Media Use	Problematic Mobile Phone Use
	OR	OR	OR
	(95% CI)	(95% CI)	(95% CI)
ACEs − 0	reference	reference	reference
ACEs – 1	1.90***	1.30	1.47***
	(1.33, 2.70)	(0.80–2.12)	(1.23–1.75)
ACEs – 2	2.31***	1.47	1.67***
	(1.63–3.30)	(0.91–2.40)	(1.39-2.00)
ACEs – 3	3.24***	1.75*	1.86***
	(2.25–4.68)	(1.04–2.96)	(1.52–2.28)
ACEs – 4+	4.93***	2.39**	2.00***
	(3.35–7.25)	(1.37–4.17)	(1.58–2.53)
**Panel B: With Confounding Variables**			
	**Problematic Video Game Use**	**Problematic Social Media Use**	**Problematic Mobile Phone Use**
	OR	OR	OR
	(95% CI)	(95% CI)	(95% CI)
ACEs − 0	reference	reference	reference
ACEs – 1	1.68**	1.18	1.44***
	(1.17–2.41)	(0.71–1.94)	(1.20–1.73)
ACEs – 2	1.91***	1.20	1.56***
	(1.32–2.75)	(0.71–1.98)	(1.30–1.89)
ACEs – 3	2.29***	1.14	1.58***
	(1.56–3.38)	(0.65-2.00)	(1.28–1.96)
ACEs – 4+	3.11***	1.52	1.57***
	(2.05–4.73)	(0.83–2.79)	(1.22–2.03)

*indicates p < 0.05,** indicates p < 0.01, *** indicates significant at < 0.001. Panel B models include age, sex, race/ethnicity, household income, parent education, site, twin, depressive, anxious, and stress symptoms. ACEs = Adverse childhood experiences; CI = Confidence Interval.

## Discussion

This large, demographically diverse, national sample of 9,673early adolescents who use screens found that a greater ACE score was significantly associated with greater problematic video game and mobile phone use (when problematic screen use was measured as a binary outcome). Youth exposed to 4 or more ACEs experienced 3.1 times higher odds of reported problematic video game use and 1.6 times higher odds of problematic mobile phone use compared to peers with no ACEs. Given that behavioral addictions can have significant negative personal, social, and professional impacts, understanding risk factors to their development is critical.

Increased rates of problematic screen use among a nationally representative study of U.S. screen-using youth with higher ACE scores builds upon prior findings. Previous studies have shown that ACEs are associated with higher video game use among Japanese adolescents [14] and Chinese university students [13] and higher mobile phone addiction in Chinese university students [18]. In addition, a 2019 study of exclusively high-risk U.S. youth found a dose-dependent relationship between ACEs and problematic media use using parent-report data [16]. This study builds upon these prior findings by using youth self-report data and showing that ACEs are an important risk factor for problematic screen use among a demographically diverse sample of U.S. early adolescents. Our study further contributes to the literature by focusing on early adolescents, who represent a critical age group because this developmental period is vulnerable to developing health-related risk factors [1, 48]. This study also found that problematic social media use, a contemporary, novel measure associated with adverse physical and mental health consequences, was not associated with ACEs in the logistic model (Table 2), though a dose-dependent relationship was observed in the linear model (Supplemental Table 3) [49, 50] It is important to note for these mixed results that rates of problematic social media use were low at3.5%, which may be due to relative younger age of the participants in our study (11–12 years old). Accordingly, it is important to continue to examine the association between ACEs and problematic screen-use in a broader sample of adolescents of older ages, during which time social media use – and the possibility of problematic social media use – may be elevated.

This study found that 7.0% of US adolescents who used screens endorsed problematic video game use and 21.8% reported problematic phone use. These rates are similar than prior studies that have found that 7.6% of German adolescents report video game addiction [51] and 22.9% of Chinese adolescents report problematic phone use; however, as our study excluded youth who did not report screen use, the true average use is likely lower; we suspect may be partially secondary to the lower average age in the ABCD cohort (11–12 years). The high rates of problematic mobile phone use, approaching one in four early adolescents, is concerning for early education around mobile phone use and behaviors.

Limitations to this study include vulnerability to confounding variables; however, given the breadth of the ABCD cohort measures we did include many covariates (sex, race/ethnicity, income, parent education, mental health conditions). In addition, the ABCD dataset does not include a single, validated scale, so this score was generated from questions across different surveys capturing the same themes as the original ACEs screener. Therefore, future research should reassess the questions posed in this study using such a scale. In addition, we analyzed data from youth who reported screen use, so findings cannot be generalized to all adolescents regardless of screen use. Also, cutoff scores for the VGAQ and SMAQ have not been clearly established in the literature and were extrapolated conservatively from the Bergen Facebook Addiction Scale. Finally, mobile phone and social media behavior may have areas of overlap (Supplemental Table 3) [52].

## Conclusions

This study demonstrates that, among adolescents who use screens, higher ACE scores are associated with problematic screen use, particularly problematic video game and mobile phone use, which has important clinical and public policy implications for screen time recommendations. For example, clinicians should be aware of the increased risk of problematic screen use among youth with high ACE scores, explore video game, social media, and mobile phone use among this population, and collaborate with families to implement a family media use plan informed by the American Academy of Pediatrics [53]. In addition, schools may consider implementing curricula focused on promoting healthy digital habits [54]. Future studies should explore protective factors to problematic video game, social media, and mobile phone use among ACE-exposed adolescents and the effectiveness of clinic-based interventions for this population.

## Electronic supplementary material

Below is the link to the electronic supplementary material.

Supplementary Material 1

## Data Availability

Data used in the preparation of this article were obtained from the ABCD Study (https://abcdstudy.org), held in the NIMH Data Archive (NDA). Investigators can apply for data access through the NDA (https://nda.nih.gov/).

## List of abbreviations

ACEs: Adverse Childhood Experiences
ABCD study: Adolescent Brain Cognitive Development
VGAQ: Video Game Addiction Questionnaire
SMAQ: Social Media Addiction Questionnaire
MPIQ: Mobile Phone Involvement Questionnaire
