# Supplementary material for: Associations between adverse childhood experiences and early adolescent problematic screen use in the United States
Source: BMC Public Health. 2023 Jun 22;23:1213. doi: 10.1186/s12889-023-16111-x (PMC10286460; doi:10.1186/s12889-023-16111-x)
Supplement: Supplementary file 1 — Supplementary Material 1 [file 12889_2023_16111_MOESM1_ESM.docx]

Supplemental Information

**Supplemental Table 1: Childhood Adverse Childhood Experiences (ACEs) Scale from the ABCD study**

| ACE Category | ABCD Question | Original ACE questionnaire^1^ - While you were growing up, during your first 18 years of life: | ABCD Assessment* |
| --- | --- | --- | --- |
| Physical abuse | Shot, stabbed, or beaten brutally by grown-up in the home | Did a parent or other adult in the household often or very often:   - Push, grab, slap, or throw something at you?   Or   - Ever hit you so hard that you had marks or were injured? (yes/no) | KSADS-5 PTSD Module – Parent |
|  | Beaten to the point of having bruises by a grown-up in the home |  | KSADS-5 PTSD Module – Parent |
| Sexual abuse | A grownup in the home touched your child in their privates, had your child touch their privates, or did other sexual things to your child | Did an adult or person at least 5 years older than you ever…   - Touch or fondle you or have you touch their body in a sexual way?   Or   - Attempt or actually have oral, anal, or vaginal intercourse with you? (yes/no) | KSADS-5 PTSD Module – Parent |
|  | An adult outside your family touched your child in their privates, had your child touch their privates or did other sexual things to your child |  | KSADS-5 PTSD Module - Parent |
| Household Violence | Witness the grownups in the home push, shove, or hit one another | Was your mother or stepmother:   - Often or very often pushed, grabbed, slapped, or had something thrown at her?   Or   - Sometimes, often, or very often kicked, bitten, hit with a first, or hit with something hard?   Or   - Ever repeatedly hit at least a few minutes threatened with a gun or knife? (yes/no) | KSADS-5 PTSD Module – Parent |
|  | Family members sometimes hit each other |  | Family Environment Scale- Parent & Youth Report |
|  | Family members sometimes get so angry they throw things |  | Family Environment Scale- Parent & Youth Report |
| Substance abuse in the household | Has any blood relative of your child ever had any problems due to alcohol such as: marital separation or divorce, laid off or fired from work, arrests or DUIs; alcohol harmed their health; in an alcohol treatment program; suspended or expelled from school 2 or more times; isolated self from family, caused arguments or were drunk a lot?** | Did you live with anyone who was a problem drinker or alcoholic or who used street drugs? (yes/no) | Family History Assessment – Parent |
| Household mental illness | Has ANY blood relative of your child ever attempted or committed suicide?** | Was a household member depressed or mentally ill, or did a household member attempt suicide? (yes/no) | Demographics survey – Parent |
|  | Has ANY blood relative of your child ever suffered from depression, that is, have they felt so low for a period of at least two weeks that they hardly ate or slept or couldn't work or do whatever they usually do?** |  | Demographics survey – Parent |
| Divorce/separation | Divorced/separated | Were your parents every divorced or separated? (yes/no) | Demographics survey – Parent |
| Criminal household member | Has ANY blood relative of your child been the kind of person who never holds a job for long, or gets into fights, or gets into trouble with the police from time to time, or had any trouble with the law as a child or an adult? | Did a household member go to prison? (yes/no) | Family History Assessment – Parent |
| Emotional neglect | Believes in showing his/her love for me*** | Did you often or very often feel that…   - No one in your family loved you or thought you were important or special?   Or   - Your family didn’t look out for each other, feel close to each other, or support each other? (yes/no) | CRPBI Acceptance Subscale – Youth |
| Physical neglect | How often do your parents/guardians know where you are?**** | Did you often or very often feel that…   - You didn’t have enough to eat, had to wear dirty clothes, and had no one to protect you?   OR   - Your parents were too drunk or high to take care of you or take you to the doctor if you needed it? (yes/no) | Parental Monitoring Survey |
|  | If you are at home when your parents or guardians are not, how often do you know how to get in touch with them?**** |  | Parental Monitoring Survey |

*All ACEs data were determined through parent and adolescent responses in the baseline (2016-2018), one-year follow-up (2017-2019), and first half of two-year follow-up (2018-2019) surveys. A yes response to any of the following nine ACEs at any timepoint was counted as one-point.

**one-point given if blood relative was mother or father

***one-point given if respondent answered “not like him/her” when describing his/her primary caregiver

****one-point if never/almost never

1. Felitti VJ, Anda RF, Nordenberg D, et al. Relationship of Childhood Abuse and Household Dysfunction to Many of the Leading Causes of Death in Adults: The Adverse Childhood Experiences (ACE) Study. *American Journal of Preventive Medicine*. 1998;14(4):245-258. doi:10.1016/S0749-3797(98)00017-8

**Supplemental Table 2:  Correlations among problematic video game, social media, and mobile phone use**

|  | 1 | 2 | 3 |
| --- | --- | --- | --- |
| Video Game Addiction Questionnaire Score | - |  |  |
| Social Media Addiction Questionnaire Score | 0.44** | - |  |
| Mobile Phone Involvement Questionnaire Score | 0.34** | 0.59** | - |

**p < 0.001

**Table 3. Associations among ACE score and problematic video game use, social media use, and phone use scores (N=11,875)**

| **Panel A: Bivariate Model** | | | |
| --- | --- | --- | --- |
| **Problematic screen use measures** | **Problematic Video Game Use** | **Problematic Social Media Use** | **Problematic Mobile Phone Use** |
|  | B | B | B |
|  | (95% CI) | (95% CI) | (95% CI) |
| ACEs - 0 | reference | reference | reference |
| ACEs – 1 | 0.19*** | 0.11** | 0.06*** |
|  | (0.13 - 0.27) | (0.05- 0.18) | (0.03 - 0.08) |
| ACEs – 2 | 0.28*** | 0.15*** | 0.08*** |
|  | (0.21- 0.35) | (0.07- 0.21) | (0.05- 0.11) |
| ACEs – 3 | 0.37*** | 0.18*** | 0.10*** |
|  | (0.29 - 0.46) | (0.10- 0.26) | (0.07- 0.13) |
| ACEs – 4+ | 0.53*** | 0.30*** | 0.11*** |
|  | (0.43 -0.63) | (0.20-0.40) | (0.07- 0.15) |
| **Panel B: With Confounding Variables** | | | |
|  | **Problematic Video Game Use** | **Problematic Social Media Use** | **Problematic Mobile Phone Use** |
|  | B | B | B |
|  | (95% CI) | (95% CI) | (95% CI) |
| ACEs - 0 | reference | reference | reference |
| ACEs – 1 | 0.13** | 0.10** | 0.05*** |
|  | (0.06- 0.19) | (0.03- 0.17) | (0.02- 0.08) |
| ACEs – 2 | 0.17*** | 0.11** | 0.06*** |
|  | (0.11- 0.24) | (0.04-0.18) | (0.04- 0.09) |
| ACEs – 3 | 0.20*** | 0.10* | 0.07*** |
|  | (0.12- 0.28) | (0.01- 0.18) | (0.03- 0.10) |
| ACEs – 4+ | 0.32*** | 0.19*** | 0.07** |
|  | (0.22- 0.41) | (0.09- 0.29) | (0.02- 0.11) |
| *indicates p<0.05, ** indicates statistical significance at p<0.01, *** indicates statistical significance at p<0.001. Panel B models include age, sex, race/ethnicity, household income, parent education, site, twin, depressive, anxious, and stress symptoms. ACEs = Adverse childhood experiences; CI = Confidence Interval. | | | |

**Figure 1. Adolescent respondents included in problematic video game, social media, and mobile phone use models**


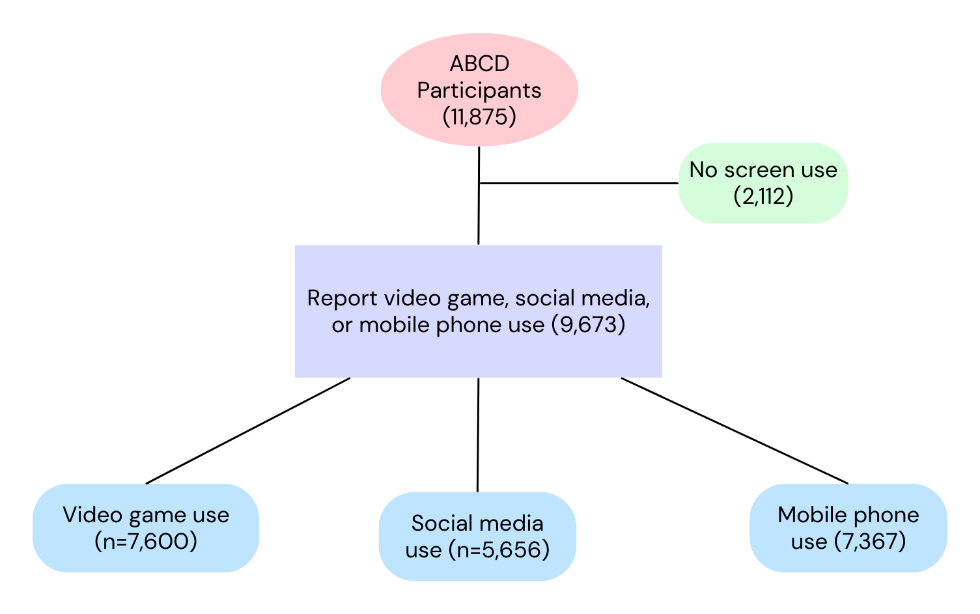


**Figure 2. Problematic screen use questionnaire scores (video game, social media, mobile phone) by reported ACE score**
